# Resting-state posterior alpha power changes with prolonged exposure in a natural environment

**DOI:** 10.1186/s41235-020-00247-0

**Published:** 2020-10-27

**Authors:** Rachel J. Hopman, Sara B. LoTemplio, Emily E. Scott, Ty L. McKinney, David L. Strayer

**Affiliations:** 1 Center for Cognitive and Brain Health, Department of Psychology, Northeastern University, 805 Columbus Ave, 670 ISEC, Boston, MA 02115 USA; 2 Department of Psychology, University of Utah, 380 S. 1530 E. RM 502, Salt Lake City, UT 84112 USA

**Keywords:** Attention restoration theory, Cognitive neuroscience, Electrophysiology, Electroencephalography, Environmental effects, Cognitive processes

## Abstract

Exposure to environments that contain natural features can benefit mood, cognition, and physiological responses. Previous research proposed exposure to nature restores voluntary attention – attention that is directed towards a task through top down control. Voluntary attention is limited in capacity and depletes with use. Nature provides unique stimuli that do not require voluntary attention; therefore, the neural resources needed for attention to operate efficiently are theorized to restore when spending time in nature. Electroencephalography reflects changes in attention through fluctuations in power within specific frequencies. The current study (*N* = 29) measured changes in averaged resting state posterior alpha power before, during, and after a multiday nature exposure. Linear mixed-effects models revealed posterior alpha power was significantly lower during the nature exposure compared to pre-trip and post-trip testing, suggesting posterior alpha power may be a potential biomarker for differences related to exposure to natural and urban environments.

## Significance statement

Previous research shows that sedentary indoor lifestyles promote negative health outcomes and induce cognitive fatigue. However, exposure to natural environments, like parks or greenspaces, can restore cognitive functioning and improve overall mood. Research has yet to determine the underlying neural mechanisms related to exposure in natural environments. This study uses electroencephalography collected during rest to examine changes in neurophysiological indices of attention after prolonged exposure in nature. We provide evidence for the neurological changes during exposure to natural environments. This applied approach informs our understanding of relationship between environmental exposures and neuroelectric functioning.

## Introduction

Nature provides unique visual and auditory stimuli that benefit mood and cognitive performance. Nature – defined in this context as non-manmade ecosystems that support a rich diversity of vegetation and complex views – are rated as optimal for restoration of cognitive processing (Stigsdotter, Corazon, Sidenius, Refshauge, & Grahn, 2017). Unpredictable and spacious environments were associated with higher self-reported creativity (van Rompay & Jol, 2016), and naturalist gardens without elements of structure are perceived more restorative compared to formal, structured gardens (Twedt, Rainey, & Proffitt, 2016). Walking in nature improved positive affect (Hartig, Evans, Jamner, Davis, & Gärling, 2003) and decreased self-reported anxiety, rumination, and negative affect (Bratman, Daily, Levy, & Gross, 2015). Similarly, regularly viewing nature correlated with higher self-reported mood (Tennessen & Cimprich, 1995), especially nature scenes containing water (Felsten, 2009). Aesthetics of the natural world are consistently reported to increase perceived restoration of cognition and improve mood (Kaplan & Berman, 2010).

Prior research also shows exposures to nature improve performance on tasks measuring working memory, executive functioning, and creative performance (Ohly et al., 2016). Performance on the backwards digit span and operation span task (Bratman et al., 2015) improved after a brief walk through nature but not an urban environment and persisted 30 min after the walk (Gidlow et al., 2016). In addition, creative performance increased by 50% after spending a prolonged time in a natural environment (Atchley, Strayer, & Atchley, 2012). Passively viewing scenes of nature, in contrast to urban environments, also improved performance on a sustained attention task (Li & Sullivan, 2016) and boosted measures of voluntary attention and inhibition (Tennessen & Cimprich, 1995).

Research has yet to determine the specific mechanisms that drive the interaction between cognitive processing and environmental exposure. Kaplan and Kaplan (1989) suggested that voluntary attention is depleted through persistent, daily use in a typical urban environment built with multiple streams of information that bombard attention. Voluntary attention – attention that is required to ignore distractors and focus on a task at hand – is difficult to maintain over time (MacLean et al., 2009). Because neural resources used to direct attention during a task are limited in capacity (Posner, Snyder, & Davidson, 1980), the ability to sustain voluntary attention depletes with use (Parasuraman, Warm, & See, 1998). Exposure to natural environments can restore depleted attentional resources by downregulating voluntary attention and increasing involuntary attention – awareness of the environment without mental preparation to act on it (Kaplan, 1995). When employed under conditions of rest, involuntary attention uses bottom-up mechanisms to process the environment (Folk, Remington, & Johnston, 1992). According to the Attention Restoration Theory (ART; Kaplan, 1995), natural environments present stimuli that promote involuntary attention while simultaneously decreasing voluntary attention, allowing for restoration to occur.

ART (Kaplan, 1995) defines several characteristics of natural, non-manmade environments that are thought to promote involuntary attention and therefore restore voluntary attention. ART proposes that in order for attention to be restored, the environment must be complex enough to captivate externally-focused attention (i.e. *extent*). The environment must also *be away* from mental or physical distractions associated with a cognitively-demanding environment, such as notifications and reminders that pull attention away from the environment. The individual should find the environment *compatible* with their preferences, as well contain *soft fascinations,* or visual characteristics that intrigue attention. Kaplan (1995) proposes that environments that contains these features – such as natural, non-manmade environments – promote involuntary, externalized attention and therefore restore attentional processing.

The neurophysiological processes underlying cognitive improvements from nature exposure have yet to be determined. In attempts to measure neural activity while immersed in nature, several studies have used portable electroencephalography (EEG) to determine changes in neurophysiological responses from short exposures in nature. For example, Chen, He, and Yu (2016) collected EEG for a 20-min period while participants were exposed to a real-world nature or urban environment, with the former showing higher global EEG correlation between electrodes in the right hemisphere. Greater correlated activity potentially suggests improved neural processing (Chen et al., 2016), although more research is needed to determine the relationship between global EEG signals and neural functioning. Norwood et al. (2019) provide a review of other recent work that use EEG and functional magnetic resonance imaging (fMRI) measures during brief nature exposures. While urban exposures were associated with increased activity in regions associated with voluntary attention, such as increased prefrontal cortex activity, natural environments were associated with increased theta-band signals and decreased neural activity in frontal, voluntary attention regions (Norwood et al., 2019). Because most research has collected EEG during brief exposures in nature, more research is needed to investigate the relationship between neural oscillatory processes and longer exposures to nature using conventional laboratory EEG recording systems.

Research has yet to determine how fluctuations in EEG activity relate to changes in externalized and internalized attention from exposure to natural environments. Resting posterior alpha (PA) power is one potential biomarker of fluctuations in attention. Simultaneous fMRI and EEG measurements collected during rest showed PA power positively correlated with signals in neural regions associated with introspection (Bowman et al., 2017) and negatively correlated with neural regions associated with vigilance and visual processing (Laufs, Kleinschmidt, et al., 2003), suggesting higher PA power is indicative of internally focused attention (Bowman et al., 2017). Similarly, prior research shows lower resting PA power correlated with narrower attentional breadth on a subsequent attention task, suggesting lower PA power indicates greater focus on local details in the environment (Pitchford & Arnell, 2019). During eyes-opened resting conditions, the visual system actively processes visual stimuli and PA power, as a neural inhibitory mechanism, is typically low (Foxe & Snyder, 2011). During eyes-closed conditions the visual cortex is not actively processing the environment, resulting in increased PA power (Zou et al., 2009). Whenever PA power temporarily decreased during eyes-closed rest, neural signals in the fronto-parietal cortices increased, indicative of increased external awareness of the environment (Laufs, Krakow, et al., 2003). Collectively, this research suggests that fluctuations in PA power could reflect shifts in attention during both eyes-opened and closed conditions.

The alpha oscillation is thought to be generated from postsynaptic potentials in a cingulo-insular-thalamic network associated with attentional alertness (Sadaghiani et al., 2010). This network, comprised of the dorsal anterior cingulate cortex, anterior insula, anterior prefrontal cortex, and thalamus, is proposed to maintain tonic internalized alertness (Coste & Kleinschmidt, 2016) and may correlate with fluctuations in involuntary attention (Dosenbach, Fair, Cohen, Schlaggar, & Petersen, 2008). Previous research suggests that the thalamus regulates shifts in attention during rest when eyes are opened or closed (Portas et al., 1998). The alpha oscillation is proposed to be an inhibitory mechanism generated by a thalamic-cortical loop, with higher PA power inhibiting external awareness of the environment (Foxe & Snyder, 2011) and reductions in PA power occurring when attention is externally directed, such as towards attention-grabbing stimuli in the environment (Kirschfeld, 2005). Therefore, higher resting PA power potentially indicates lower external awareness, whereas lower PA power during rest indicates higher awareness of the environment.

Nature contains specific visual stimuli that are often rated as more fascinating and restorative than an urban environment (Berto, Massaccesi, & Pasini, 2008). Fascinating sensory features or other characteristics within a natural environment that are not present in an urban environment may cause attention to be externalized. We hypothesized resting PA power would significantly decrease when participants were in a natural environment compared to an urban environment during both eyes-opened and closed conditions. This study measured within-subject neuroelectric changes during rest to determine if prolonged time in nature (i.e. a 4-day nature trip) influenced neuroelectric power. Rather than directly interpreting fluctuations in PA power as fluctuations in attentional processing, we seek to determine if exposure to environmental exposures have a unique influence on neuroelectric power during rest. In these analyses, we measured differences in power during eyes-opened and eyes-closed resting conditions in both natural and urban environments using a pre-during-post repeated measures design.

## Methods

### Participant demographics

**Fig. 1:**
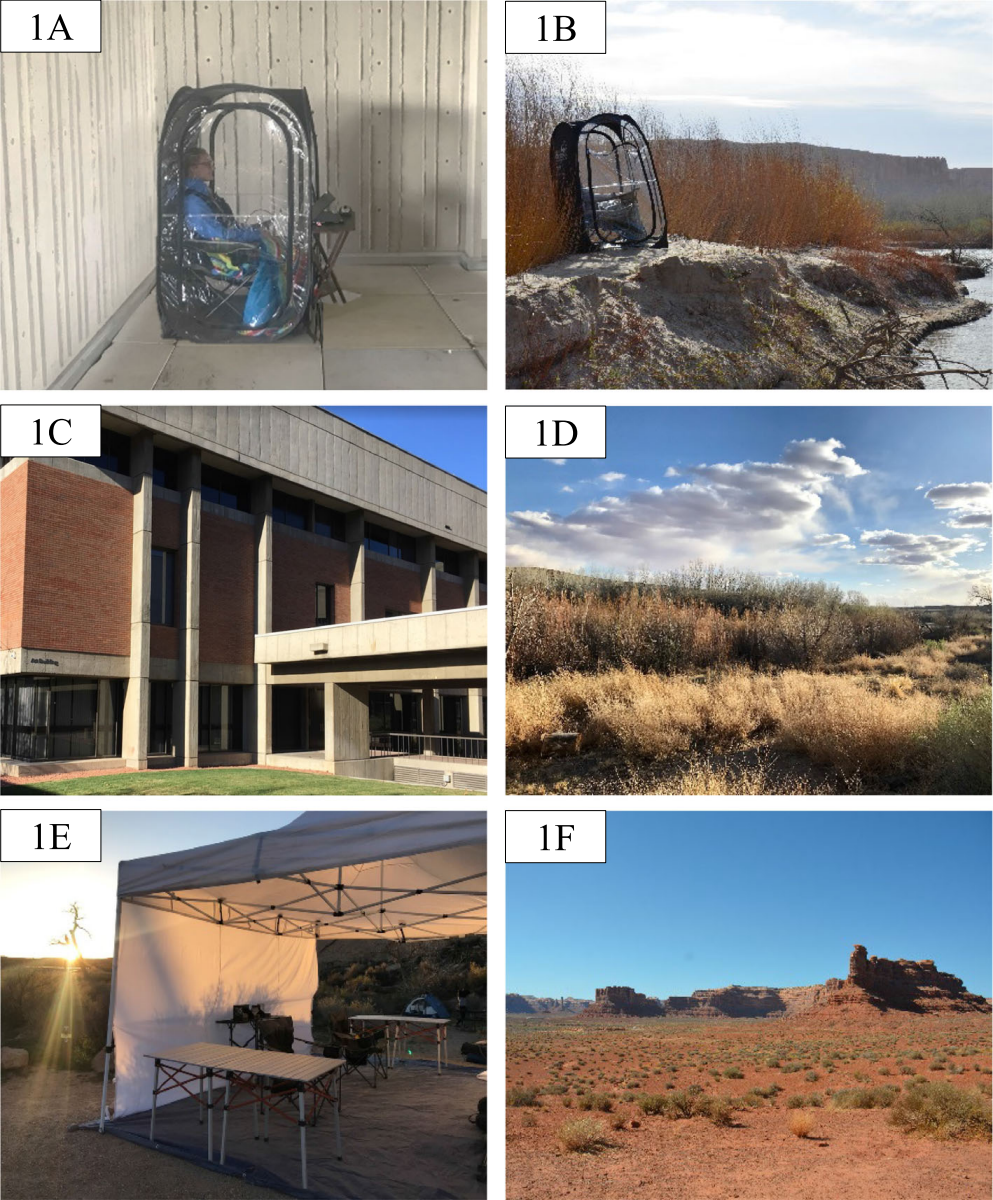
Example images from the research study. **a**: Example of participant in weather-protecting pod in the pretrip and post-trip setting. **b**: Example of participant in weather-protecting pod in the nature trip setting. **c**. Example view of pre-trip and post-trip environment. **d**. Example view of nature trip environment. **e**. EEG preparation setup at the nature trip. **f**. San Rafael Swell hiking area

Twenty-nine participants (9 M, 19 F, 1 O; mean age: 25.48) completed testing before, during, and after a 4-day nature trip in Bluff, UT. Six participants’ data were removed from all sessions due to inadequate data collection because of issues stemming from the amplification hardware during the online recording session, and one participant’s data were removed due to not following instructions during data collection. Ten participants had data from two of the three sessions removed due to excess artifacts in the eyes-opened condition. Nine participants had one session removed from the eyes-opened condition and eight participants had one session removed from the eyes-closed condition due to excess artifacts. Twenty-two participants’ data were used in the final analyses for the eyes-opened condition and 28 participants’ data were used in the eyes-closed condition. Participants were recruited from and volunteered to participate in association with a pre-organized trip associated with a university course offered during Spring 2018. During each day of the trip, participants engaged in low-to-moderate intensity hiking in the San Rafael Swell region, a high desert country with landscapes of red rock mesas rising above sandy desert terrain (see Fig. 1f). Participants hiked at their chosen pace for a 4-h period (less than 4 miles a day), in combination with frequent breaks as part of the class trip. Once the daily hiking concluded, participants returned to camp in the afternoon and either completed the trip testing session or engaged in chosen activities (i.e. walking around campground, resting, journaling). All received monetary compensation for their participation.

### Procedure

During each testing session, researchers prepared the participant for the EEG recording by exfoliating the skin surface using NuPrep abrasive gel applied using a cotton swab where electrooculogram electrodes would be positioned. The skin was then rinsed with water and 10 mm diameter Ag/AgCl biopotential electrodes were placed over the mastoids, the lateral canthi of both eyes, and above and below the left eye using adhesive electrode collars and filled with saline based gel. The head was scratched using a comb and hair was parted to allow for greater contact between the electrode cap and skull. A Neuroscan 40-channel cap was then fitted to the head and positioned to the correct alignment. The electrodes built in the EEG cap are configured based on the International 10–20 system (Jasper, 1958). Researchers assured that impedances were below 10 kΩ for each electrode by inserting a saline solution into the QuikCell sponges. Electrode AFz – preassigned in the cap – was used as the ground and electrode A1 was used as the online reference. EEG data were collected using Curry 8.0 software on a 64-bit Windows 10 laptop and recorded using a Neuroscan NuAmp amplifier. Data were sampled online at a rate of 1000 Hz. Participants’ field of view and range of motion were not impeded when wearing the EEG cap, but participants were instructed to limit eye movements during all recording sessions.

During all testing periods, participants sat quietly in a remote, outdoor location while keeping their eyes opened for a five-minute period and then eyes closed for a five-minute period. Participants received a brief break between eyes-opened and eyes-closed conditions for the researcher to check impedance levels. Participants sat along a riverbank during the recording period for trip testing (Fig. 1c) and outside among buildings on campus during pre-trip and post-trip testing (Fig. 1d). To control for weather-related disturbances in the data (such as wind, rain, or sun), participants sat on a chair in a weather-protected clear plastic pod during all sessions. Participants’ field of view and range of motion were not impeded when in the pod as the clear plastic was translucent. The pod kept the participant and equipment dry and protected from weather during each session (Fig. 1a and b). Differences in weather-related factors between pre-, trip, and post-trip testing sessions are reported in Table 1. Once the recording was complete, participants completed other behavioral and subjective tasks before the EEG cap was removed and participants were debriefed.

**Table 1:** Averaged weather reported from Salt Lake City International Airport, UT and Four Corners Regional Airport, UT during data collection in April 2018

	Place	No. Days	Avg high (°F)	Avg low (°F)	Avg Sun	Avg humidity (%)
Pretrip	Salt Lake City, UT	6	62.4	54.4	Partly Cloudy	33.4
Trip	Bluff, UT	3	61.3	55.3	Sunny	13.3
Post-Trip	Salt Lake City, UT	10	67.5	61.5	Partly Cloudy	23.8

*Note*: Weather data collected from timeanddate.com/weather from 12 pm–6 pm during respective data collection days

### Processing pipeline

Data were first highpass filtered at 0.1 Hz using filt.m function in MATLAB toolbox EEGlab (Delorme & Makeig, 2004) to remove drift noise and re-referenced to the average of the mastoids using reref.m. Electrodes in regions of interest were kept for the remaining analyses (midfrontal power: FP2, F3, Fz, F4, and FPz; posterior power: O1, Oz, O2, PO1, and PO2). Electrodes within the region of interest that exhibited poor signal-to-noise ratio were interpolated using eeg_interp.m (if more than one electrode required interpolation, the file was then removed). Data was segmented into 1-s epochs across the first 5-min period and epochs containing movement or blink artifacts were removed.^1^ Average power across the alpha band (8–12 Hz) was extracted using a Fast Fourier Transform with a Hanning window and was log-transformed for data analysis. Average log-transformed power across the theta band (4–8 Hz) was also extracted as a comparison in broadband changes. Log-transformed power was averaged across epochs, resulting in a separate estimate for each band and electrode. Electrodes in midfrontal regions were reduced separately from electrodes in posterior regions.

## Results

Linear mixed effects models were used to compare within subject differences across sessions using lme4 package version 1.17 R software 3.5.1 (Bates, Mächler, Bolker, & Walker, 2015). These models used a random intercept to account for differences in baseline resting power between participants and maximum likelihood to estimate mean change across the three sessions. Linear mixed models were selected because they adjust for sources of non-independence (multiple time points from the same participant) and allow for an unbalanced design with missing data. Session was used to predict differences in PA power over the three sessions. The sessions were dummy coded to compare differences in power between urban (pre-trip and post-trip testing) and nature testing sessions (trip testing), as well as differences between pre-trip and post-trip sessions. Additional models compared pre to trip testing and trip testing to post-trip testing sessions. Post hoc exploratory analyses included gender as a predictor for all models. R package lmertest (Kuznetsova, Brockhoff, & Christensen, 2017) was used to calculate degrees of freedom and *p*-values using Satterthwaite approximation.

### Eyes-opened condition

**Fig. 2:**
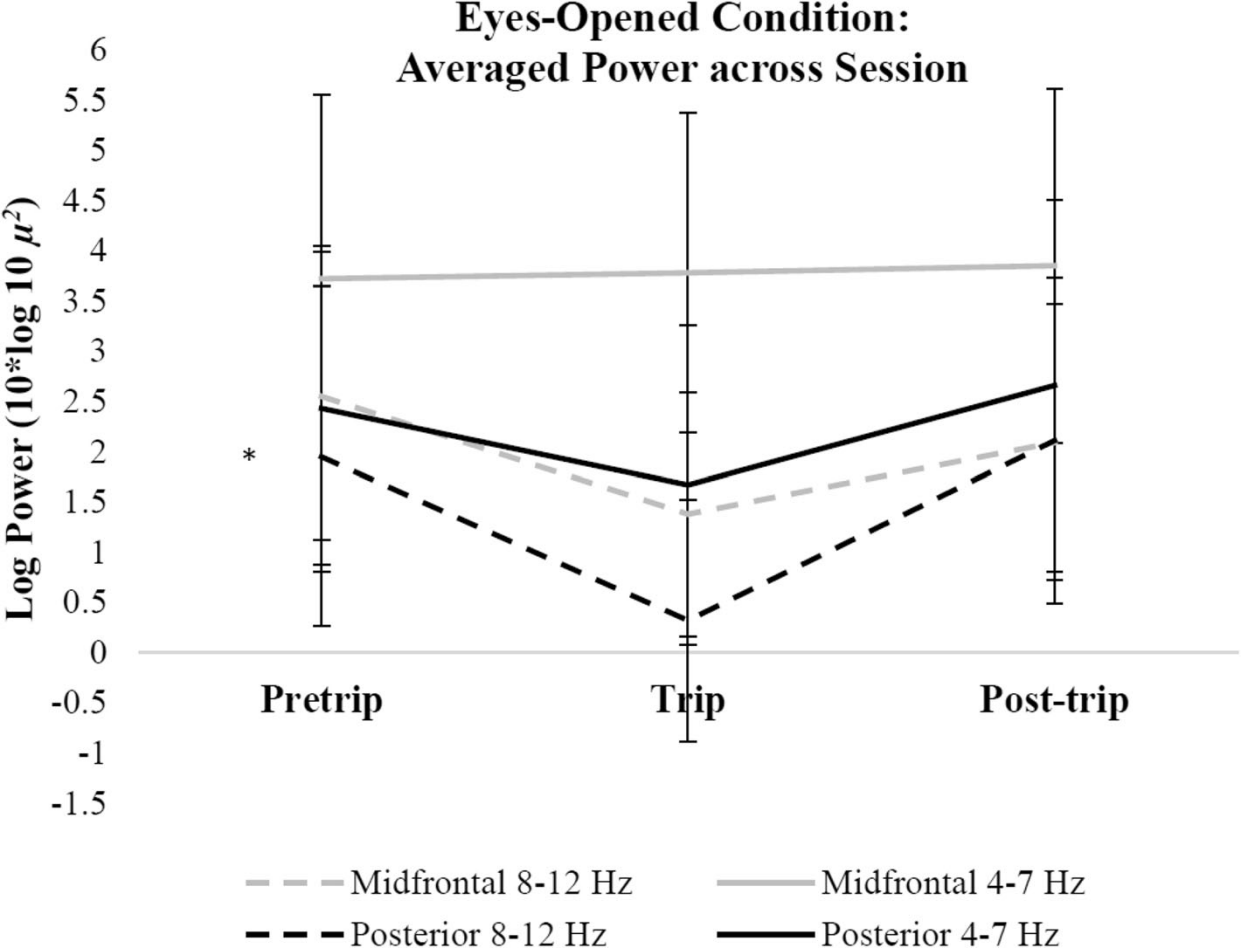
Changes in log transformed power across sessions during the eyes-opened condition. Error bars indicate 95% confidence intervals around the mean. * *p* < 0.01

**Fig. 3:**
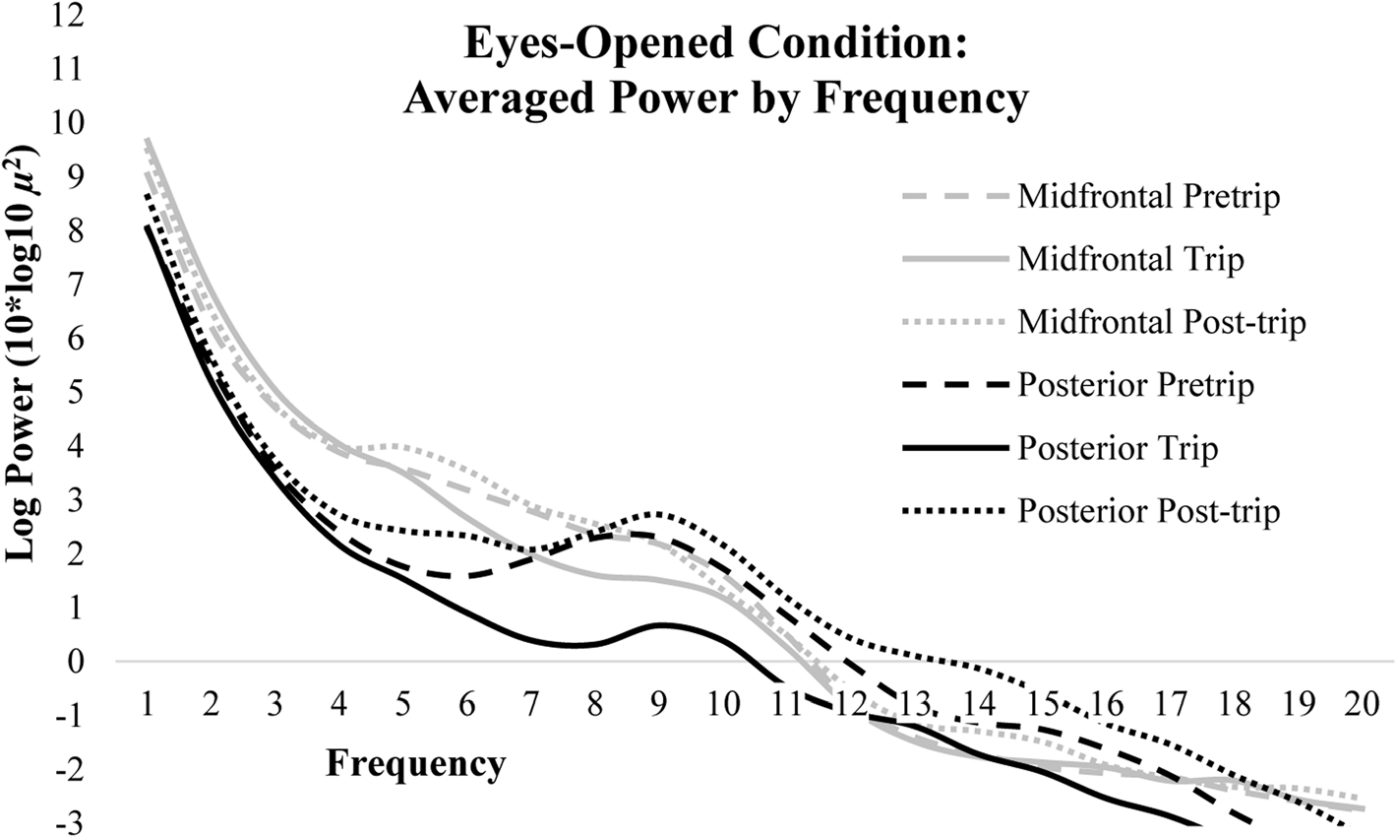
Spectra for each session averaged across midline and posterior electrodes during the eyes-opened condition

Linear mixed models revealed that eyes-opened PA (8–12 Hz) power was significantly lower during the nature compared to urban exposures (*p* < 0.05). PA power did not differ between post and pretrip sessions (*p* > 0.05; Fig. 2). Differences in log-transformed power across sessions were isolated to PA power, as alpha and theta power in the midfrontal region-of-interest and theta power in the posterior region-of-interest were not significantly different during the nature exposure (trip session) compared to urban exposures (pre and post-trip sessions, *p* > 0.05) or during the post compared to pretrip session (*p* > 0.05; Fig. 3). See Table 2 for all eyes-opened linear mixed model comparisons of log transformed power for both frontal and posterior regions-of-interest in alpha power and power within 4–8 Hz.

**Table 2:** Regression coefficients for session comparisons regressed onto electrode sites for power collected during eyes-opened rest

	Estimate (*β*)	95% CI		df	*t*	*p*
Posterior alpha power						
(Intercept)	1.59	0.21	2.98	20.82	2.30	0.03*
Nature vs urban	−1.77	−3.12	− 0.43	33.81	−2.62	0.01*
(Intercept)	1.62	0.21	3.03	20.80	2.30	0.03*
Pretrip vs post-trip	0.32	−0.90	1.54	33.64	0.53	*NS*
(Intercept)	1.59	0.20	2.98	20.80	2.29	0.03*
Pretrip vs trip	−1.10	−2.98	0.07	33.60	−1.85	0.07
(Intercept)	1.61	0.22	3.01	20.83	2.32	0.03*
Trip vs post-trip	1.54	0.37	2.72	33.93	2.61	0.01*
Midfrontal alpha power						
(Intercept)	2.14	0.93	3.35	20.64	3.52	< 0.01*
Nature x urban	−0.79	−2.28	0.66	34.16	−1.07	*NS*
(Intercept)	2.14	0.92	3.36	20.69	3.50	< 0.01*
Pretrip vs post-trip	−0.35	−1.60	0.89	33.84	−0.56	*NS*
(Intercept)	2.13	0.92	3.34	20.65	3.52	< 0.01*
Pretrip vs trip	−0.74	−1.99	0.49	33.80	−1.18	*NS*
(Intercept)	2.14	0.93	3.37	20.65	3.52	< 0.01*
Trip vs post-trip	0.42	−0.87	1.73	34.38	0.64	*NS*
Posterior theta power						
(Intercept)	2.36	1.32	3.40	21.00	4.53	< 0.001**
Nature vs urban	−0.78	−2.03	0.44	34.45	−1.26	*NS*
(Intercept)	2.37	1.32	3.43	21.02	4.51	< 0.001**
Pretrip vs post-trip	0.27	−0.78	1.32	34.13	0.52	*NS*
(Intercept)	2.36	1.31	3.40	21.00	4.51	< 0.001**
Pretrip vs trip	−4.42	−1.48	0.62	34.11	−0.80	*NS*
(Intercept)	2.37	1.33	3.41	21.01	4.54	< 0.001**
Trip vs post-trip	0.75	−0.32	1.83	34.53	1.39	*NS*
Midfrontal theta power						
(Intercept)	3.93	2.92	4.95	20.94	7.73	< 0.001**
Nature vs urban	0.30	−0.74	1.34	34.04	0.58	*NS*
(Intercept)	3.93	2.92	4.95	20.98	7.75	< 0.001**
Pretrip vs post-trip	0.15	−0.73	1.03	33.82	0.33	*NS*
(Intercept)	3.93	2.92	4.95	20.98	7.75	< 0.001**
Pretrip vs trip	0.29	−0.59	1.16	33.79	0.66	*NS*
(Intercept)	3.93	2.91	4.95	20.96	7.74	< 0.001**
Trip vs post-trip	−0.15	−1.07	0.77	34.20	−0.33	*NS*

*Note*: Individual eyes-opened linear mixed model comparisons of log transformed power for both frontal and posterior regions-of-interest in alpha power and power within 4–8 Hz

* *p* < 0.05. ** *p* < 0.01

### Eyes-closed condition

**Fig. 4:**
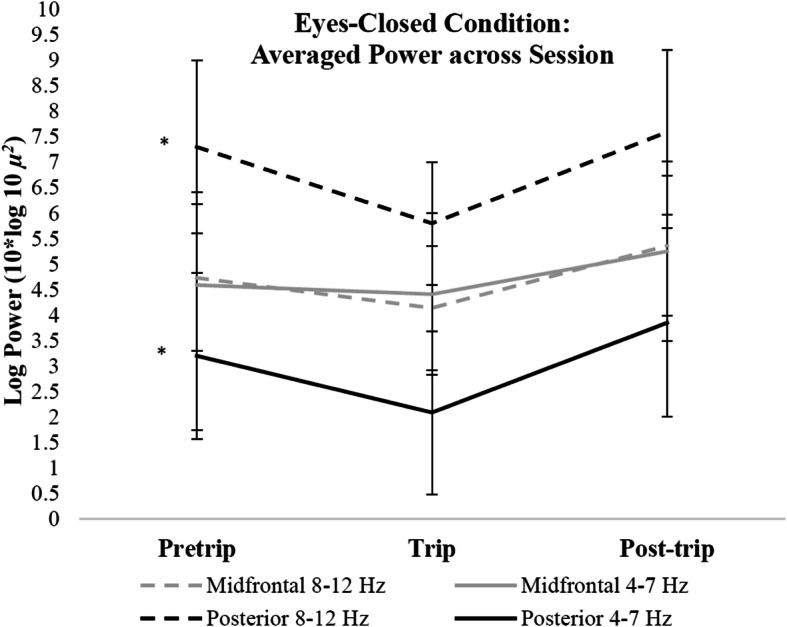
Changes in log transformed power across sessions during the eyes-closed condition. Error bars indicate 95% confidence intervals around the mean. * *p* < 0.01

**Fig. 5:**
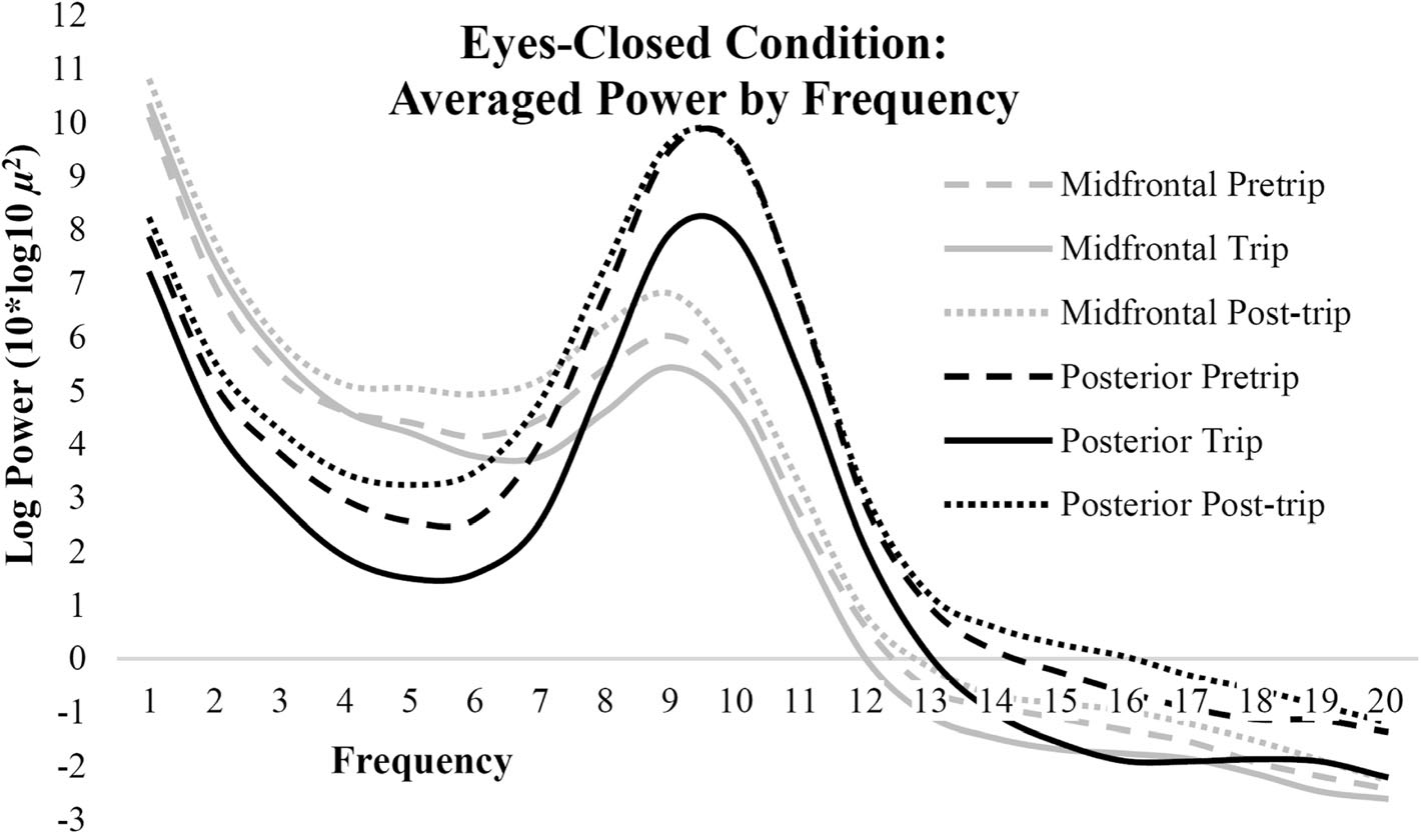
Spectra for each session averaged across midline and posterior electrodes during the eyes-closed condition

Linear mixed models revealed eyes-closed log transformed PA (8–12 Hz) power was also significantly lower during the nature compared to urban exposures (*p* < 0.05; Fig. 4). Posterior theta power (*p* < 0.01) and midfrontal alpha power (*p* < 0.05) was also significantly lower during the nature compared to urban exposures. PA and posterior theta power did not differ between pre and post-trip sessions (*p* > 0.05), whereas, midfrontal alpha and theta power were higher during post versus pretrip (*p* < 0.05) and trip (midfrontal alpha: *p* < 0.01; midfrontal theta: *p* < 0.05) sessions. Differences in log transformed power across 2–20 Hz for each session during the eyes-closed condition are plotted in Fig. 5. See Table 3 for all eyes-closed mixed model comparisons.

**Table 3:** Regression coefficients for session comparisons regressed onto electrode sites for power collected during eyes-closed rest

	Estimate (*β*)	95% CI		df	*t*	*p*
Posterior alpha power						
(Intercept)	7.05	5.45	8.66	26.82	8.76	< 0.001**
Nature vs urban	−1.82	−2.85	− 0.80	47.27	−3.51	0.001*
(Intercept)	7.13	5.52	8.75	26.73	8.81	< 0.001**
Pretrip vs post-trip	0.24	−0.65	1.14	46.87	0.54	*NS*
(Intercept)	7.08	5.47	8.69	26.79	8.78	< 0.001**
Pretrip vs trip	−1.15	−2.07	−0.24	47.18	−2.50	0.02*
(Intercept)	7.07	5.47	8.68	26.80	8.77	< 0.001**
Trip vs post-trip	1.43	0.56	2.32	47.17	3.23	< 0.01*
Midfrontal alpha power						
(Intercept)	4.87	3.64	6.10	26.74	7.87	< 0.001**
Nature vs urban	−0.93	−1.89	0.01	47.39	−1.95	0.06`
(Intercept)	4.91	3.67	6.15	26.69	7.88	< 0.001**
Pretrip vs post-trip	0.80	0.07	1.53	46.84	2.16	0.04*
(Intercept)	4.90	3.67	6.14	26.70	7.90	< 0.001**
Pretrip vs trip	−0.20	−1.03	0.62	47.24	− 0.48	*NS*
(Intercept)	4.86	3.63	6.10	26.75	7.84	< 0.001**
Trip vs post-trip	1.13	0.36	1.89	47.21	2.91	< 0.01*
Posterior theta power						
(Intercept)	3.20	2.08	4.33	26.94	5.66	< 0.001**
Nature vs urban	−1.50	−2.58	−0.43	47.93	−2.77	< 0.01*
(Intercept)	3.26	2.13	4.40	26.84	5.72	< 0.001**
Pretrip vs post-trip	0.52	−0.36	1.41	47.12	1.17	*NS*
(Intercept)	3.23	2.10	4.37	26.90	5.68	< 0.001**
Pretrip vs trip	−0.77	−1.71	0.17	47.74	−1.61	*NS*
(Intercept)	3.20	2.08	4.33	26.92	5.69	< 0.001**
Trip vs post-trip	1.37	0.48	2.26	47.68	3.03	< 0.01*
Midfrontal theta power						
(Intercept)	4.87	3.98	5.76	27.05	10.90	< 0.001**
Nature vs urban	−0.21	−1.13	0.70	48.20	−0.45	*NS*
(Intercept)	4.88	3.99	5.78	27.00	10.88	< 0.001**
Pretrip vs post-trip	0.74	0.05	1.42	47.26	2.13	0.04*
(Intercept)	4.89	4.00	5.79	27.00	10.87	< 0.001**
Pretrip vs trip	0.28	−0.49	1.04	47.87	0.73	*NS*
(Intercept)	4.85	3.97	5.74	27.07	10.92	< 0.001**
Trip vs post-trip	0.57	−0.18	1.33	47.94	1.49	*NS*

*Note*: Individual eyes-closed linear mixed model comparisons of log transformed power for both frontal and posterior regions-of-interest in alpha power and power within 4–8 Hz

* *p* < 0.05. ** *p* < 0.01

### Eyes-closed versus eyes-opened comparisons

**Fig. 6:**
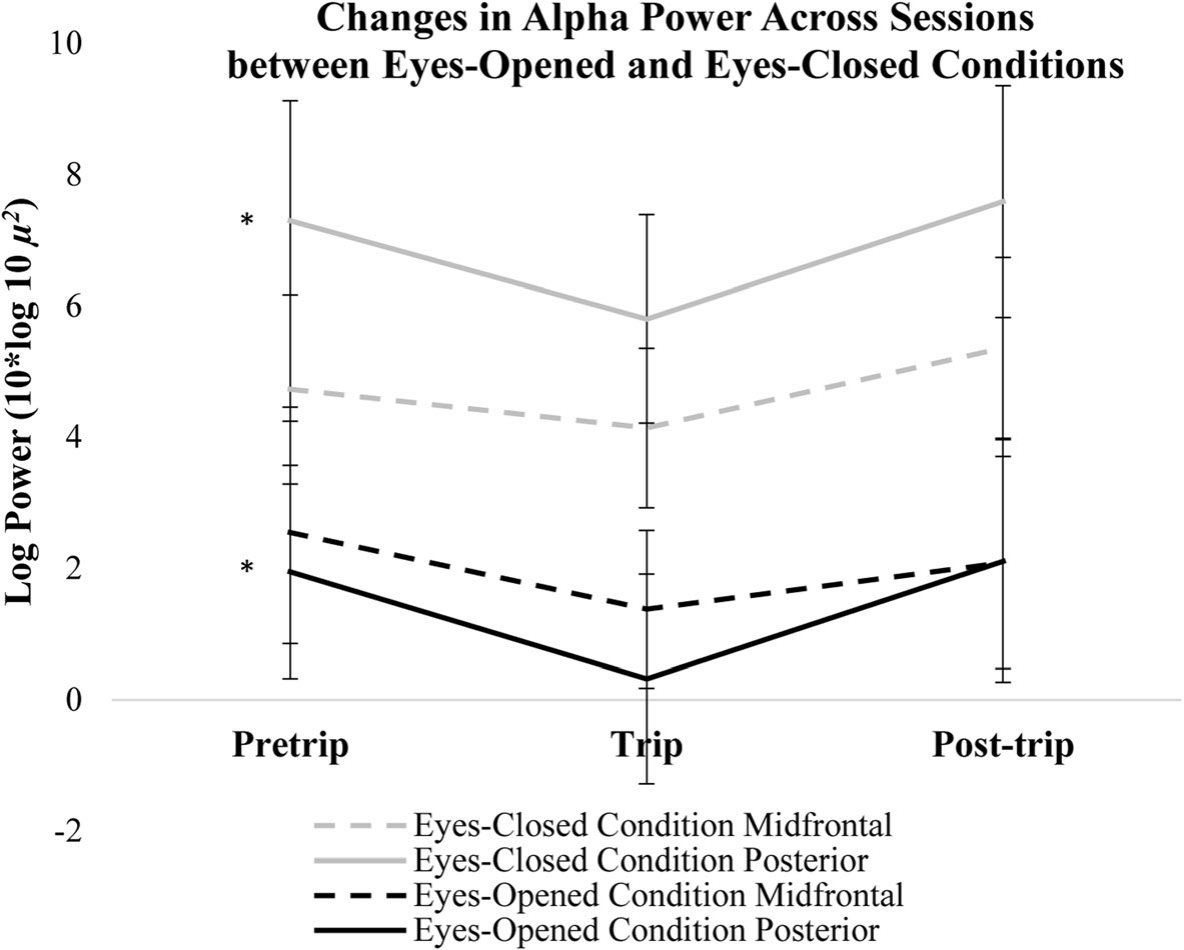
Changes in log transformed alpha power (8–12 Hz) across sessions during the eyes-opened and closed conditions. Error bars indicate 95% confidence intervals around the mean. * *p* < 0.01

**Fig. 7:**
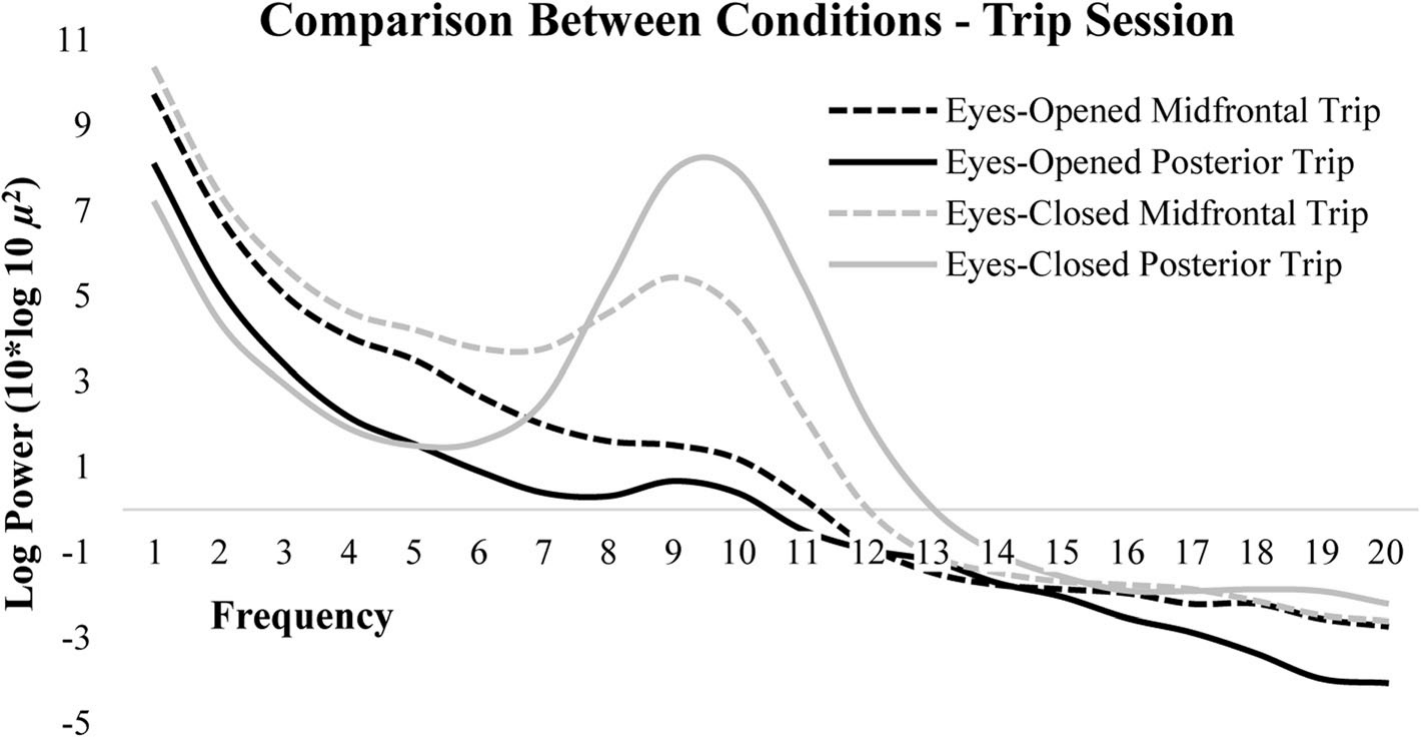
Log transformed spectra for the trip session averaged across midline and posterior electrodes during the eyes-opened and eyes-closed conditions

Paired sample t-tests were used to compare differences in PA and midfrontal alpha power between eyes-opened and eyes-closed conditions for each session. Paired sample t-tests revealed that alpha power in both midfrontal (Pretrip: *t*(45) = 4.17, *p* < 0.05; Trip: *t*(1,38) = 11.08, *p* < 0.01; Post-trip: *t*(44) = 11.27, *p* < 0.01) and posterior (Pretrip: *t*(47) = 20.31, *p* < 0.001; Trip: *t*(38) = 21.17, *p* < 0.001; Post-trip: *t*(47) = 22.53, *p* < 0.001) regions-of-interest were significantly higher in the eyes-closed conditions compared to eyes-opened conditions for all sessions (Fig. 6). Paired sample t-tests were also used to compare differences between posterior and midfrontal power within the eyes-opened and eyes-closed conditions. PA power was significantly higher than midfrontal alpha power during the eyes-closed condition across sessions (Pretrip: *t*(52) = − 2.28, *p* < 0.05; Trip: *t*(42) = − 1.73, *p* < 0.05; Post-trip: *t*(52) = − 2.05, *p* < 0.05), but midfrontal and posterior alpha power did not significantly differ during the eyes-opened condition across sessions (Pretrip: *t*(40) = 0.50, *p* > 0.05; Trip: *t*(34) = 1.58, *p* > 0.05; Post-trip: *t*(39) = 0.26, *p* > 0.05). Log transformed spectral power during the trip session across midfrontal and posterior regions-of-interest for both eyes-opened and eyes-closed conditions is plotted in Fig. 7.

### Post-hoc analyses: differences in posterior alpha power based on gender

Emerging evidence suggests environmental exposures show differential effects on cognition based on gender (Izenstark & Ebata, 2016). Therefore, post-hoc analyses were conducted to determine differences based on gender in PA power across sessions.^2^ Linear mixed models revealed overall eyes-opened PA power was marginally lower for females compared to males (B = − 2.77, *t*(18.4) = − 1.92), *p* = 0.07), however, males and females did not significantly differ in eyes-opened PA power across sessions (*p* > 0.05). Similarly, linear mixed models revealed overall eyes-closed PA power did not differ based on gender (*p* > 0.05) and males and females did not significantly differ in eyes-closed PA power across sessions (*p* > 0.05).

## Discussion

Nature is proposed to provide uniquely fascinating stimuli that capture involuntary attention, thereby allowing voluntary attention to rest and restore (Kaplan, 1995). As a result, time spent in natural environments has been shown to benefit cognitive functioning (Atchley et al., 2012; Berto, Baroni, Zainaghi, & Bettella, 2010; Tennessen & Cimprich, 1995), physiological stress responses (Laumann, Gärling, & Stormark, 2003), and mood (Bratman et al., 2015; Hartig et al., 2003). Previous research shows prolonged exposure to nature results in changes to cognitive functioning (Atchley et al., 2012); however, research has yet to determine if prolonged exposure to natural environments relates to changes in neuroelectric signatures related to attention. Previous research suggests fluctuations in PA power reflect differences in attentional processing – higher PA power is thought to reflect increased internal processing and rumination (Bowman et al., 2017) whereas lower PA power reflects increased external processing (Laufs, Krakow, et al., 2003) and vigilance (Liu et al., 2012). This study used a repeated-measures design to determine within-person changes in neural signals during rest when exposed to urban and natural environments. Participants completed three sessions of eyes-opened and eyes-closed resting EEG before, during, and after a multiday nature trip.

As hypothesized, PA power was significantly lower during the nature exposure compared to pretrip and post-trip testing for both the eyes-closed and eyes-opened conditions. Given the consistency of the current trip’s environment to the key features proposed by ART – being away, extent, soft fascination, and compatibility – this finding suggests possible neural correlates of the restoration process. The nature trip provided participants the opportunity to be away from daily distractions, as well as provided diverse, interesting stimuli to capture attention toward the environment throughout the trip. Stimuli in natural environments influence allocation of involuntary attention during rest (Fan, McCandliss, Fossella, Flombaum, & Posner, 2005); therefore, lower resting PA power may indicate greater attention towards the environment during the nature trip compared to pre-trip and post-trip.

In agreement with previous research, PA power was higher during the eyes-closed compared to eyes-opened conditions for all sessions. Because alpha power in visual processing areas can reflect spatial awareness of the environment (Klimesch, 1999), eliminating visual attention increases alpha power in the visual cortex (Kirschfeld, 2005) through increased thalamic activity (Liu et al., 2012). Although the visual features in the environment were not actively processed by the visual cortex during the eyes-closed condition, differences in PA power between the nature and urban sessions may suggest that attention was externally focused during the nature trip. However, the natural environment presented different sensory features than the urban environment, which may have also altered PA power during eyes-closed rest. Regardless, significant differences in neuroelectric power isolated to the PA frequency exist between the natural and urban testing environments.

Changes in PA power may potentially relate to other factors besides fluctuations in attentional processing, such as lower level visual perceptual processes. For example, differences in luminance, auditory features, or the number of visual features between the nature and urban environments may have also produced changes in PA power during rest. Previous research shows viewing fascinating scenes of nature with more visual features have lasting benefits to cognitive performance whereas viewing scenes with fewer fascinating features did not show the same effect (Berman, Jonides, & Kaplan, 2008; Berto et al., 2010). Improvements in cognitive performance from viewing scenes of nature suggest that visual qualities within the natural and urban environment influence cognitive functioning, and potentially alter neuroelectric signals of attention. While the eyes-closed condition controlled for visual differences between testing environments, the lasting benefits from viewing nature may have influenced neuroelectric signals during the eyes-closed condition. However, differences in PA power may have resulted from other undocumented differences that are not related specifically to the nature or urban environment, such as differences in recording quality or unique sensory information in the environments. Although all sessions were completed outdoors using identical methods, the nature environment may have introduced other factors that could explain differences in neuroelectric power unrelated to attentional processing.

Although fluctuations in resting PA power broadly relate to attention, the precise mechanisms of this relationship remain unknown. Other recent research also shows event-related potentials related to performance on cognitive tasks are altered during prolonged exposure to nature (LoTemplio et al., 2020) and increased activity in higher frequency ranges (beta; 14–30 Hz) while viewing contemplative, natural landscapes (Olszewska-Guizzo, Paiva, & Barbosa, 2018); therefore, other neuroelectric markers may also relate to environmental exposures. Because global PA power is a rudimentary measure of attention, future concurrent task-based EEG studies using refined measures of attentional processing could help elucidate the specific underlying neural circuits of the restoration effect.

Unexpectedly, alpha power at midfrontal sites and posterior theta power showed a significant decrease during the nature exposure during the eyes-closed condition, suggesting that overall power was reduced during the trip testing during the eyes-closed condition. Likewise, both midfrontal and posterior regions showed overall higher power across frequencies during post-trip testing for the eyes-closed condition. These differences in power were not reflected in the eyes-opened condition. Despite differences in overall power between conditions, PA power significantly decreased during the trip testing compared to pre-trip and post-trip testing for both eyes-opened and closed conditions. Post-hoc analyses revealed change in PA power across sessions did not significantly differ based on gender. However, the sample size for this study had significantly more females than males. More research with larger sample sizes is necessary to understand potential gender differences in resting PA power from environmental exposures.

A limitation of the current study is the lack of comparison between prolonged time in nature compared to other environments. Future research should compare the effects seen from prolonged time in nature to a non-nature trip to determine if natural environments have differential effects on neural functioning. This comparison would control for other potential factors associated with time away from daily living. Because the current findings were from a multiday nature trip, future experiments could also explore if changes in PA power replicate from shorter durations in nature. More work is needed to determine the rate at which PA power returns to baseline upon return to urban environments and collect larger samples to investigate individual differences that potentially moderate the neural differences associated with environmental exposures. Collectively, such studies could argue for an ‘ideal dose’ of nature for cognitive restoration and an understanding of the mediating neural circuitry.

## Conclusions

This research is the first to show prolonged time in nature relates to fluctuations in neural biomarkers and the first to compare changes in resting spectral power over the course of several weeks when exposed to different environments. Overall, these findings suggest prolonged exposure to nature is associated with decreased PA power compared to time in urban environments. Spending prolonged time in nature may alter neural signatures that relate to the tendency to focus on internal thoughts and increase awareness of the external environment, but fluctuations in neuroelectric signals could be indicative of other potential neural processes. In any event, this research shows evidence that prolonged environmental exposures uniquely influence neuroelectric power during rest. Future research can expand upon this work to understand how neuroelectric fluctuations relate to prolonged exposure in natural and urban environments.

## Data Availability

The datasets used and/or analyzed during the current study are available from the corresponding author on reasonable request.

## Abbreviations

ART: Attention restoration theory
EEG: Electroencephalography
PA: Posterior alpha

## Footnotes

1 *Percent Rejected:* Frontal Eyes-Opened: 45.71%; Posterior Eyes-Opened: 44.21%, Frontal Eyes-Closed: 26.92%, Posterior Eyes-Closed: 24.82%

2 Gender “Other” (*N* = 1) was excluded from analyses.
